# 2529. Population pharmacokinetic models for cefepime and enmetazobactam derived from pooled Phase 1 to Phase 3 clinical studies

**DOI:** 10.1093/ofid/ofad500.2147

**Published:** 2023-11-27

**Authors:** Jannik Vollmer, Adam Belley, Patrick Velicitat, Matthias Machacek

**Affiliations:** LYO-X AG, Basel, Basel-Stadt, Switzerland; Allecra Therapeutics SAS, Beaconsfield, Quebec, Canada; Allecra Therapeutics SAS, Beaconsfield, Quebec, Canada; LYO-X AG, Basel, Basel-Stadt, Switzerland

## Abstract

**Background:**

The investigational β-lactam/β-lactamase inhibitor combination of cefepime (FEP)/enmetazobactam (ENM) met criteria for non-inferiority and superiority compared to piperacillin/tazobactam in a phase 3 clinical trial of adult patients with complicated urinary tract infections (cUTI)/acute pyelonephritis (AP). In this study, a population pharmacokinetic (PK) model is presented that describes FEP and ENM plasma PK in adult cUTI/AP patients.

**Methods:**

Plasma PK samples were analyzed from participants receiving FEP (n=588) and ENM (n=649) in three phase 1 (n=132), and single phase 2 (n=43) and phase 3 (n=488) clinical trials. Population PK parameters were assessed and the relationship between different covariates and model parameters were investigated. The effect of infection on disposition was assessed by testing disease covariates on fixed and random effects for all PK parameters.

**Results:**

A two-compartment model with linear clearance of FEP (5.95 L/h) and ENM (7.68 L/h) from the central compartment best described the PK of both compounds. Common covariates for both compounds were de-indexed estimated glomerular filtration rate (eGFR), age, and body weight while ENM PK covariates also included cUTI infection and sex. Simulations showed that the only covariate requiring dose adjustment was renal function. The final model simultaneously described FEP and ENM plasma PK, which enabled estimates of the correlation of the random effects between PK parameters. Clearances and volumes of distribution (Vd) were strongly correlated within individuals between the two compounds (correlation coefficients >0.9). Variability was higher in cUTI patients although differences in mean ENM or FEP exposures between healthy subjects and cUTI patients were negligible. The total Vd was 16.9 L for FEP and 20.6 L for ENM. Clearance of both agents was found to depend on de-indexed eGFR (covariate coefficients ≥0.83), suggesting that renal filtration is the predominant clearance mechanism. The terminal elimination half-life was 2.2 h for FEP and 2.0 h for ENM.

Final population PK model population parameter estimates for cefepime and enmetazobactam determined from the pooled phase 1 to phase 3 clinical studies

**Figure:**
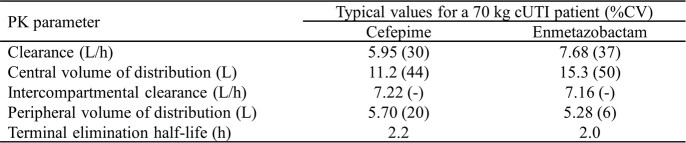

**Conclusion:**

The population PK models for FEP and ENM predict exposures in cUTI patients, including subjects with renal impairment. These models support Monte-Carlo simulations and target attainment estimations in adult cUTI/AP patients.

**Disclosures:**

**Adam Belley, PhD**, Allecra Therapeutics SAS: Advisor/Consultant **Patrick Velicitat, MD**, Allecra Therapeutics SAS: Salary

